# Comparison of Bioabsorbable Magnesium versus Titanium Screw Fixation for Modified Distal Chevron Osteotomy in Hallux Valgus

**DOI:** 10.1155/2018/5242806

**Published:** 2018-11-19

**Authors:** Baver Acar, Ozkan Kose, Adil Turan, Melih Unal, Yusuf Alper Kati, Ferhat Guler

**Affiliations:** University of Health Sciences, Antalya Training and Research Hospital, Department of Orthopedics and Traumatology, Antalya, Turkey

## Abstract

**Objective:**

The purpose of this retrospective study was to compare the clinical and radiological results of magnesium versus titanium screw fixation for modified distal chevron osteotomy in hallux valgus (HV).

**Materials and Methods:**

A total of 31 patients who underwent modified distal chevron osteotomy for HV deformity between 2014 and 2017 were reviewed retrospectively. Headless magnesium (Mg) compression screw fixation was applied in 16 patients (17 feet) and headless titanium (Ti) compression screw in 15 patients (17 feet). Patients were followed up for at least 12 months with a mean of 19.0 ± 6.8 months in the Mg screw group and 16.2 ± 6.19 in the Ti screw group, respectively (p: 0.234). Clinical results were evaluated using the American Orthopedic Foot and Ankle Society Hallux metatarsophalangeal-interphalangeal (AOFAS-MTP-IP) scale and a visual analogue scale (VAS). The hallux valgus angle (HVA) and intermetatarsal angle (IMA) were measured before and after surgery. Time to osteotomy union and any complications were recorded and compared between the groups.

**Results:**

An improvement in the AOFAS-MTP-IP scale and VAS points were recorded in both groups with no statistically significant difference between the groups (p: 0.764 and 0.535, resp.). At the final follow-up examination, HVA and IMA were similar (p: 0.226 and 0.712, resp.). There was no significant loss of correction between the early and final radiographs in respect of HVA and IMA in both groups (p: 0.321 and p: 0.067). Full union of the osteotomy was obtained in all patients. Prolonged (1.5 months) swelling and mild hyperemia around the surgical incision were observed in 1 patient in the Mg group but there was a good response to physical and medical therapy, and the complaints were completely resolved. There were no other significant complications in either group.

**Conclusion:**

The results of this study showed that bioabsorbable Mg compression screw fixation has similar therapeutic efficacy to Ti screw fixation in respect of functional and radiological outcomes. Bioabsorbable Mg screw is an alternative fixation material that can be safely used for modified distal chevron osteotomy in HV surgery.

## 1. Introduction

Hallux valgus (HV) is a common foot deformity that may cause pain, disability, difficulty in shoe wear, and gait disturbance. Initially, a period of conservative care can be tried but if the symptoms are not relieved and the deformity has progressed, surgical correction is usually necessitated. More than 300 surgical procedures have been described over the years for the correction of HV deformity [1]. The decision-making process for HV surgery depends on the severity of the deformity determined by the clinical findings and radiological measurements. Currently, distal metatarsal chevron osteotomy is commonly used for the correction of mild-to-moderate HV deformities with a congruent metatarsophalangeal (MTP) joint [2].

The technique for a distal chevron osteotomy was initially described by Austin and Leventen without performing an osseous fixation [3]. These authors thought that the three-dimensional configuration of the osteotomy was inherently stable and additional medial capsulorrhaphy provided further stability. Subsequently several modifications have been made to the technical part of the procedure, including the angle of the osteotomy and the use of various alternative methods of internal fixation [4]. The reason behind these modifications was to decrease the rates of nonunion, avascular necrosis of the metatarsal head, and malunion and to prevent postoperative loss of reduction, thereby allowing early rehabilitation and weight-bearing [5].

Metallic screw fixation is now the most frequently used fixation method for modified chevron osteotomies [6]. However, elective implant removal due to irritation of metallic screws has been reported in up to 26.9% of patients in reported series [7]. A second operation decreases patient satisfaction and increases the overall cost of the treatment. Therefore, to eliminate the need for implant removal, bioabsorbable fixation materials such as polyglycolide, polydioxanon, and poly-L-lactic acid pins have been used in foot surgery [8–10]. Although successful results have been reported with these materials, some complications such as granuloma formation and foreign body reactions, sinus formation, and allergic reactions have also been reported [11, 12].

Recently, alternative bioabsorbable screws made of magnesium (Mg) alloys have been introduced in HV surgery, particularly in the fixation of distal chevron osteotomy. Magnesium screws are produced from an alloy (MgYREZr) that contains more than 90% pure magnesium, which is naturally found in human body. When implanted in the human body, a degradation or corrosion process takes place within a certain period of time and the implant is completely absorbed and/or replaced by the native bone [13].

However, there are four clinical studies that have evaluated the efficacy of magnesium bioabsorbable screws in HV surgery [14–17], and, in three of these, the follow-up time was 6 months or less. There has been only one study that has reported long-term (3 years) clinical results of magnesium bioabsorbable screws, and, in that study, the magnetic resonance imaging (MRI) findings of only eight patients were reported at the final follow-up. Although the clinical results of these previous studies are good or excellent, there is little knowledge about radiographic assessments. The purpose of this retrospective study was to compare the clinical and radiological results of magnesium versus titanium screw fixation for modified distal chevron osteotomy in hallux valgus (HV) deformity with special interest in the radiographic findings and the degradation process.

## 2. Materials and Methods

### 2.1. Patients and Study Design

A retrospective review was made of patients who underwent modified distal chevron osteotomy for HV deformity between August 2014 and December 2017 in our hospital. Institutional review board approved the study protocol and this study was carried out in accordance with the ethical standards laid down in the 1964 Declaration of Helsinki and its later amendments (IRB approval number: 2017-134/11.07). Demographic information, clinical findings, and imaging findings were extracted from the hospital database, patient charts, medical records, operation notes, notes taken during follow-up visits, and radiological images which were stored in the picture archiving and communication system (PACS).

During the study period, a total of 45 patients were identified who underwent isolated modified chevron osteotomy with no other surgical procedures. Of these, 31 patients completed a follow-up of at least 12 months and were included in the analysis. The remaining 14 patients were either lost in follow-up (8 patients) or denied participation (6 patients) and were excluded from the study. Bioabsorbable headless Mg compression screw fixation was applied to 16 patients (17 feet) and headless titanium (Ti) compression screw to 15 patients (17 feet). Thus, the patients were separated into two groups as the Mg group and the Ti group, according to the type of screw.

### 2.2. Surgical Technique

All patients were operated on under spinal anesthesia and tourniquet control in the supine position. A medial 4-6 cm incision was made over the MTP joint, and dissection was continued down to the capsule with careful protection of the dorsal and plantar medial cutaneous nerves. A V-shaped capsular flap was created to expose the bunion and MTP joint. The medial eminence was removed at the lateral edge of the sagittal sulcus with an oscillating saw. A 90° chevron osteotomy was created, in which the plantar cut was proximal to the synovial fold, again with the oscillating saw. The capital fragment was displaced laterally (usually 2-5 mm) until sufficient correction of first ray alignment was achieved. The osteotomy was stabilized with single or double cannulated headless compression screws from the dorsum of the distal metatarsal head to the plantar shaft. The direction of screw fixation was performed in two different ways, either from the proximal fragment towards the distal fragment or from the distal fragment towards the proximal fragment according to the preference of the surgeon. A capsular flap was used to shift the proximal phalanx and was anchored to the proximal fragment with nonabsorbable sutures through a dorsal metaphyseal drill hole. The skin and subcutaneous tissues were closed in the routine manner.

### 2.3. Implants

**Figure 1:**
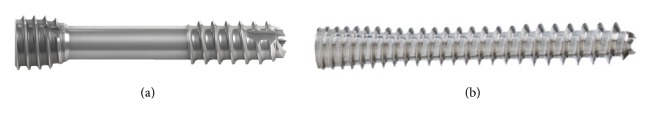
The appearance of (a) magnesium and (b) titanium screws used in the study.

Bioabsorbable Mg screws (MAGNEZIX® CS, Syntellix AG, Hanover, Germany) were used for the fixation of the osteotomy in the Mg group. It is a variable pitch cannulated headless screw (2.7 mm Ø), which provides interfragmentary compression, similar to a Herbert screw. Titanium (Alloy: Ti 6Al 4V) screws (Tasarımmed, Istanbul, Turkey) were also variable pitch cannulated headless compression screws (2.5 mm Ø), but the pitch varied continuously along the length (Figure 1).

### 2.4. Postoperative Rehabilitation

A short-leg plaster cast with the toe in anatomic alignment was applied to all the patients for two weeks. At the end of the second week, the cast was opened and the sutures were removed. Weight-bearing as tolerated was encouraged on the heel and lateral side of the foot with the patient wearing with stiff-soled shoes. MTP joint active movements were started. At the end of the sixth week, full weight-bearing with normal shoes was allowed.

### 2.5. Clinical Evaluation

Functional outcomes were assessed with the American Orthopaedic Foot and Ankle Society hallux metatarsophalangeal-interphalangeal (AOFAS-MTP-IP) scale and pain was assessed using the visual analogue scale (VAS). MTP joint range of motion (ROM, flexion plus extension) was evaluated using a goniometer and rated as normal (>75°), moderate (30°-74°), or severe (<30°) restriction. Any complications including infection, wound problems, recurrence, and revision surgery during the follow-up period were recorded.

### 2.6. Radiographic Evaluation

Hallux valgus angle (HVA) and the first and second intermetatarsal angle (IMA) were measured on preoperative, early postoperative (within the first month), and final follow-up radiographs. Measurements were taken according to the recommendations of the ad hoc committee of the American Orthopaedic Foot and Ankle Society [18]. All measurements were repeated twice by the same observer two weeks apart, and the average of the two measurements was used for the final analysis. HVA >20° on the postoperative radiographs was accepted as recurrence. Union of the osteotomy or loss of reduction and any radiographic changes such as avascular necrosis, osteolysis, and implant failure were also evaluated and recorded.

### 2.7. Statistical Analysis

Continuous variables were stated as mean ± standard deviation (SD) and categorical variables as number (n) and percentage (%). The Shapiro-Wilk test was used to assess the normality of the data, and either parametric or nonparametric tests were selected according to the distribution of each variable. The comparison of independent continuous variables was performed using Student's* t*-test and Mann-Whitney* U* test. Analyses of differences within the same group were performed using the Paired Samples* t*-test, the Wilcoxon test, and the Kruskal-Wallis test. Categorical variables were compared using the Chi-square test. A value of p<0.05 was accepted as statistically significant.

## 3. Results

### 3.1. Clinical Results

A total of 34 feet (17 in Mg group and 17 in Ti group) were analyzed. The baseline demographic and clinical characteristics of both groups were similar in respect of age, gender, side, preop HVA, preop IMA, preop VAS, preop AOFAS-MTP-IP scale, preop ROM, and follow-up duration (Table 1).

**Figure 2:**
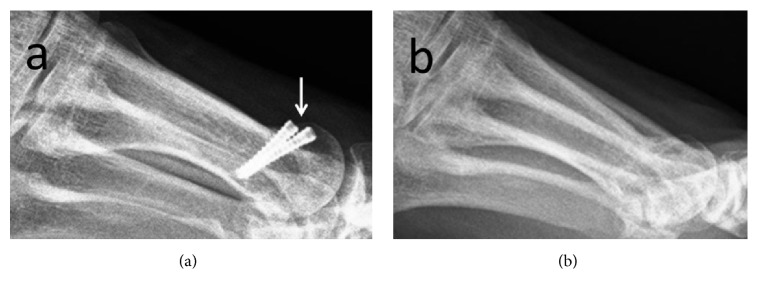
(a) Protrusion of titanium screw (*white arrow*) irritating the overlying tendons and soft tissues. (b) The symptoms were relieved after removal.

Clinically significant improvements were obtained in both groups in both the AOFAS-MTP-IP scale and VAS. In the Ti group, one patient had complaints of pain during daily activities and further examination revealed irritation of the extensor tendon by prominent screw heads. The patient's discomfort was relieved after removal of the screw at six months postoperatively (Figure 2). The rate of implant removal was statistically similar in both groups (n:1, n:0, p=0.500). In the majority of patients in both groups, the first MTP joint ROM was restricted compared to the preoperative level. At the final follow-up examination, there was no significant difference between the groups in respect of functional status (Table 2). No patients suffered from superficial or deep infection. Prolonged (1.5 months) swelling and mild hyperemia around the surgical incision were observed in 1 patient in the Mg group but there was a good response to physical and medical therapy, and the complaints were completely resolved. There were no other major complications in either group.

### 3.2. Radiographic Results

Radiological parameters (HV and IMA) were significantly improved in all patients. The correction obtained remained stable until union in both groups. No patient in either group showed significant loss of correction between the early postoperative and final follow-up radiographs. At the final follow-up, all radiographic measurements were similar in both groups (Table 3). Although the final clinical results were satisfactory (AOFAS-MTP-IP scale: 87.1 ± 8.1, VAS: 2.0 ± 2.0), HVA was measured as >20° at the final radiographic examination, which was consistent with recurrence, in 6 patients in the Mg group and 2 patients in the Ti group. Retrospective analysis of the radiographs taken immediately postoperatively demonstrated that there was initial insufficient correction rather than recurrence. The rates of recurrence were similar in both groups (p:.112). In all patients, osteotomy union was achieved within the first 3 months. The numbers of screws used for fixation (single or double) were similar in both groups (p=.500).

**Figure 3:**
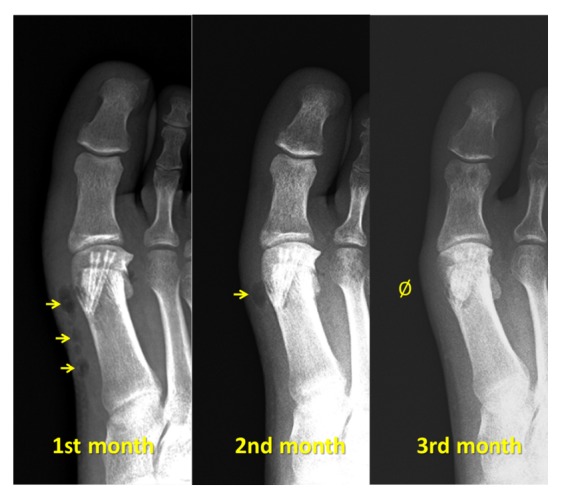
(a) Postoperative first month radiographs showing gas along the first metatarsal bone. (b) The amount of gas decreased and was limited to the metatarsal head. (b) Total absorption of gas at the third month's follow-up.

**Figure 4:**
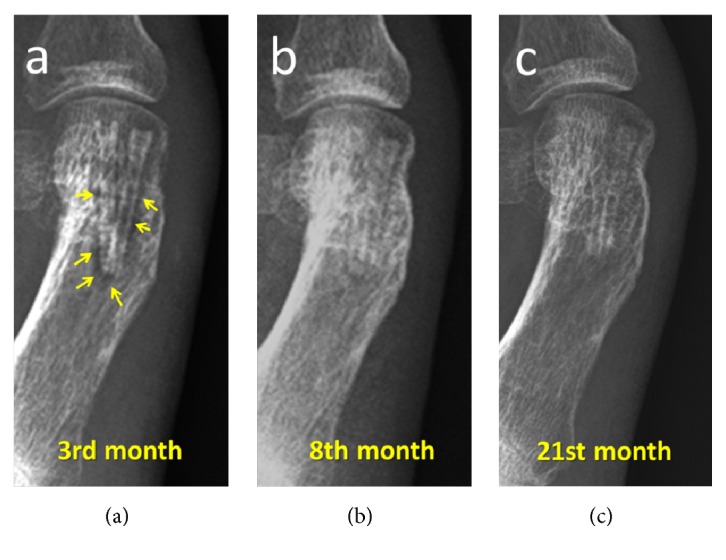
Gradual disappearance of the radiolucent zone around the screw on the 3-month (a) and 8-month (b) follow-up radiographs. (c) Complete disintegration of the Mg screws at 27 months.

In almost all patients in the Mg group (13 of 17 feet), there was a variable amount of gas accumulation within the soft tissue around the hallux during the first 2 months. After 3 months postoperatively, no gas shadows were observed within the soft tissues (Figure 3). Similarly, a radiolucent zone was observed around the screw within the bone in all patients. This radiolucency completely disappeared within 6-12 months of follow-up. These radiological phenomena were not seen to induce any clinical signs or symptoms in any patient and did not interfere with the osteotomy union. The screws were almost unidentifiable on radiographs taken 2 years postoperatively (Figure 4).

## 4. Discussion

In this retrospective study, bioabsorbable magnesium and metallic titanium headless compression screws were compared for fixation of the modified chevron osteotomy used in the treatment of HV. Similar clinical and radiological outcomes were obtained in both groups. Furthermore, the rate of complications was not significantly different between the groups. None of the patients in the Mg group and one in the Ti group (statistically insignificant) required implant removal. Magnesium bioabsorbable screws may be used as an alternative fixation method in modified chevron osteotomy, as they provide a potential advantage of a decreased rate of implant removal.

To the best of our knowledge, four previous clinical studies have reported the outcome of magnesium bioabsorbable screw fixation in HV surgery (Table 4) [14–17]. The first of these was a randomized clinical trial of 26 patients (13 Mg. versus 13 Ti. screws) who were followed up for 6 months. The clinical results were similar in the two groups without any major complications [14]. Plass et al. then reported two further studies, one as a prospective case series and the other as a randomized clinical trial [15, 16]. The results of these studies were also consistent with the first published study. More recently, Klauser reported the largest series, which was a retrospective comparison of magnesium (*n*: 100) versus titanium (*n*: 100) screw fixation for modified chevron osteotomy. The results of this large cohort showed that bioabsorbable Mg screws were not inferior to Ti screws in respect of clinical outcome and complications [17]. Including the current study, from a total of 328 patients (143 Ti versus 185 Mg) enrolled in these studies to date, there has been only one implant-related complication in magnesium screw fixation, which was the screw fracture. There were no nonunion cases. Based on the results of previous research and the current study, it is clearly obvious that Mg screws are safe and effective in the fixation of distal chevron osteotomies in hallux valgus surgery.

In addition to previous studies, this study further reports the detailed radiographic findings of the corrosion process as the patients had sequential follow-up radiographs. However, because this was a retrospective study, there were no scheduled radiographs within the same time intervals. Some unique changes were detected in the radiological follow-up that was seen in almost all patients. First, gas evolution starts immediately after implantation of magnesium screws, so, during the first two months, variable amounts of gas can be observed in soft tissues. This finding may be interpreted as a gas-producing infection similar to gas gangrene, and surgeons may be anxious about this appearance. However, this gas is quickly absorbed and is not observed later than the third month. Although Windhagen et al. did not mention this phenomenon, gas within the soft tissues has been observed by other previous authors [14–17]. Similar to soft tissues, a radiolucent zone was observed around the screws in all (100%) the current study patients on the early postoperative radiographs. In contrast to these findings, Klauser reported that this radiolucent zone was seen in only 40% of cases [17]. Plaass et al. reported the presence of a radiolucent zone around the implant in all but one of a patient series at 6 weeks and in 78% of cases at 12 weeks after surgery [16]. However, in the current series, this radiolucency continued long after 6 months even until 12 months with a decrease in size. As the osteotomy was already united, the presence of this radiolucent zone did not cause any displacement of the osteotomy.

When the metallic magnesium is exposed to body fluids, particularly the water, magnesium atoms can transfer two electrons each to water molecules. As a result of this chemical reaction, gaseous hydrogen, divalent magnesium cations, and two hydroxide ions are produced and released to the surrounding tissues. Because the hydrogen is poorly soluble in biological liquids, the gas readily diffuses away [19]. This is the basic explanation of this gas phenomenon, which can be detected with radiographic imaging. In fact, this reaction provides the bioabsorbable property to these implants and it is the normal corrosion process of magnesium implants. Controlling this reaction, in other words, adjusting the rate of corrosion is important for the clinical use of these biomaterials. If the corrosion occurs too quickly, the implant may lose its initial strength before the bone union is achieved. The implants used in this study are made of MgYREZr alloy. Based on the results obtained in this clinical study, the corrosion rate seems to be sufficiently controlled with this alloy because the bone union is achieved without displacement during the follow-up. Further reduction of the gas formed within a certain period of time should be the next goal in the development of new-generation magnesium implants. Although currently unavailable for commercial use, various coatings are under investigation such as bioceramics [20, 21]. Razavi et al. developed a composite coating composed of diopside, bredigite, and fluoridated hydroxyapatite on the AZ91 Mg alloy in order to moderate the degradation of magnesium implants. They reported decreased rate of degradation and improved bioactivity of AZ91 Mg alloy substrate; furthermore, no considerable deterioration in the compression strength was observed for the coated samples compared to the uncoated sample after 4 weeks' immersion [21]. Probably, in the near future, alternative corrosion-resistant magnesium alloys or coatings will help to fine tune the rate of biodegradation in magnesium biomaterials.

In this study, no superficial or deep infection developed in any patient of either group. Previous studies have only reported 2 cases of deep infection in patients with magnesium screw fixation. It has been shown that corrosion products of the magnesium increase the pH of the surrounding media, and this alkalization inhibits bacterial growth in vitro [19]. This could explain the low rates of infection observed to date. In contrast, in some experimental animal studies, it has been suggested that magnesium implants promote bacterial growth in vivo [22, 23]. Rahim et al. showed that biofilm layer is formed on the magnesium implants infected with* Staphylococcus aureus* and* Pseudomonas aeruginosa* strains similar to conventional steel and titanium implants [23]. In human clinical applications, these implants are applied under aseptic conditions in the operating room, but surgeons should be cautious against the possibility of infection. As with all other surgeries performed with conventional metallic implants, routine antibiotic prophylaxis should also be continued in magnesium implant operations.

The need to remove metal implants is not uncommon in foot surgery due to weak soft tissue support around the foot bones, close anatomic proximity of overlying tendons, and external pressure from shoes. Prominent screw heads or plates may irritate the skin and cause pain and discomfort when wearing shoes. In literature, implant removal rates in chevron osteotomy operations vary between 2.3% and 23.6% [7, 24, 25]. In the current series, one patient in the Ti group required removal of the screws, which corresponds to a 5.8% implant removal rate and is in line with the current literature.

One of the major advantages of magnesium screws is the elimination of the need for implant removal. None of the patients in the Mg group required implant removal even if the screw head had been left protruding. It can be considered that although the screw removal rate is very low for Ti screws, the initial higher cost of magnesium screws cannot be justified. However, even decreasing this low implant removal rate saves a significant amount of money lost in removal operations (both direct and indirect costs). Klauser performed an economic analysis of magnesium-based implants in HV surgery in Germany with the assumption that 8% of implants would require removal in 2015. According to this analysis, the money spent during the removal of the implants (direct costs) plus loss of working days (indirect costs) was calculated to be approximately 9 million Euros [17]. Therefore, when the cumulative cost of the whole treatment is taken into consideration, use of bioabsorbable Mg screw fixation may be cost-effective and may alleviate the burden of healthcare expenditure.

There are some strengths and limitations of this study. The inclusion of a small number of patients and retrospective study design are two important limitations. However, both groups were similar in terms of several preoperative demographic and clinical characteristics. All patients were followed up for at least one year, up to 32 months, with both radiographic and clinical assessment. The follow-up duration was deemed to be sufficient for the emergence of several implant-related complications.

## 5. Conclusion

In conclusion, bioabsorbable Mg screw fixation is an appropriate fixation technique for distal metatarsal modified chevron osteotomy in HV surgery, as the therapeutic efficacy and complication rates were seen to be comparable with titanium screw fixation. Moreover, implant removal operations were avoided. Unlike other bioabsorbable biomaterials, Mg screws create distinct radiological findings during degradation or the corrosion process. Both surgeons and radiologists should be familiar with these images for proper interpretation, since it can neither be called “loosening” nor infection nor does it affect bone healing.

## Figures and Tables

**Table 1:** Demographic characteristics of the patients (F: female, M: male, R: right, L: left, HVA: hallux valgus angle, IMA: intermetatarsal angle, VAS: visual analogue scale, AOFAS-MTP-IP: American Orthopaedic Foot and Ankle Society hallux metatarsophalangeal-interphalangeal scale, ROM: range of motion).

**Variable**	**Magnesium group**	**Titanium group**	**Significance**
	**(n: 16, #foot: 17)**	**(n: 15, #foot: 17)**	
**Age **	49.9 ± 15.1	48.5 ± 14.6	0.796

**Sex**	2 M /14 F	2 M / 13 F	0.675

**Side (right /left)**	7 R /10 L	10 R / 7 L	0.247

**Preop HVA**	31.0 ± 5.9	30.1 ± 6.2	0.675

**Preop IMA**	12.7 ± 1.8	12.1 ± 2.3	0.424

**Preop VAS**	4.7 ± 2.1	5.1 ± 1.9	0.620

**Preop AOFAS-MTP-IP scale**	71.2 ± 8.3	69.2 ± 9.4	0.505

**Preop ROM**			0.360

***Normal***	10	12	

***Minimal***	7	5	

***Severe***	0	0	

**Follow-up (months)**	19.0 ± 6.8	16.2 ± 6.19	0.234

MTP joint ROM (dorsiflexion plus plantar flexion), **N**: normal (>75°), **M**: minimal restriction (30°-74°), and **S**: severe restriction (<30°).

**Table 2:** Summary of radiographic results (vs.: versus).

**HVA**	**Preop**	**Early postop**	**Final**	**Significance**
**Magnesium**	31.0 ± 5.9	18.7 ± 6.3	19.6 ± 5.8	Pre vs. early vs. final: 0.000
				Early vs. final: 0.321

**Titanium**	30.1 ± 6.2	17.3 ± 4.3	17.4 ± 4.2	Pre vs. early vs. final: 0.000
				Early vs. final: 0.605

**Significance**	0.675	0.454	0.226	

**IMA**	**Preop**	**Early postop**	**Final**	**Significance**

**Magnesium**	12.7 ± 1.8	7.8 ± 2.2	8.2 ± 2.3	Pre vs. early vs. final: 0.000
				Early vs. final: 0.067

**Titanium**	12.1 ± 2.3	8.5 ± 1.9	8.0 ± 1.8	Pre vs. early vs. final: 0.000
				Early vs. final: 0.058

**Significance**	0.424	0.337	0.712	

**Table 3:** Summary of functional results.

	**Preop VAS**	**Final VAS**	**Significance**
**Magnesium**	4.7 ± 2.1	1.9 ± 1.9	.000

**Titanium**	5.1 ± 1.9	2.0 ± 0.5	.000

**Significance**	0.620	0.535	

	**Preop AOFAS-MTP-IP**	**Final AOFAS-MTP-IP**	

**Magnesium**	71.2 ± 8.3	84.1 ± 9.6	.000

**Titanium**	69.2 ± 9.4	83.0 ± 11.7	.000

**Significance**	0.505	0.764	

	**Preop ROM**	**Final ROM**	

	***N M S***	***N M S***	

**Magnesium**	10 7 0	4 10 3	.0290

**Titanium**	12 5 0	2 12 3	.0100

**Significance**	0.360	0.654	

MTP joint ROM (dorsiflexion plus plantar flexion), **N**: normal (>75°), **M**: minimal restriction (30°-74°), and **S**: severe restriction (<30°).

**Table 4:** List of previously reported clinical studies on the use of magnesium screws in modified chevron osteotomy (RCT: randomized clinical trial, SF-36: Short Form-36, VAS: visual analogue scale, NRS: numeric rating scale, FAAM: foot and ankle ability measurement).

**Author**	**Year**	**Study design**	**# patients**	**Outcome measures**	**Follow-up** **(mean)**	**Rate of implant** **removal**	**Complications**
**Windhagen H et al. [14] **	2013	RCT (Mg. vs. Ti.)	26 (13/13)	AOFAS-MTP-IP	6 months	1 in Ti. group (7.7%)	None

**Plass C et al. [15]**	2016	Prospective case-series (Mg. group only)	45	AOFAS-MTP-IP NRS FAAM SF-36	n: 39, 6 weeks n: 23, 12 weeks n:8, >26 weeks	None	1 relapse 1 hallux varus

**Plass C et al. [16]**	2017	RCT (Mg. vs. Ti.)	26 (13/13)	AOFAS-MTP-IP FAAM SF-36 VAS	3 years	1 in Ti. group (7.7%)	None

**Klauser H. [17]**	2018	Retrospective comparison (Mg. vs. Ti.)	200 (100/100)	Clinical findings	Mg. group: 12.2 weeks Ti. group: 11.7 weeks	None	Soft tissue irritation: 1 vs. 0 Delayed wound healing: 4 vs. 3 Deep infection: 1 vs. 2 Screw fracture: 0 vs. 1

**Current study**	2018	Retrospective comparison (Mg. vs. Ti.)	31 (16/15)	AOFAS-MTP-IP VAS	17.6 months	1 in Ti. group (5.8%)	Prolonged swelling: 1 vs. 0

## Data Availability

The data used in this study is stored and is available in our institutions' clinical data base and picture archiving and communications (PACS).

## References

[1] Chandler L. M. (2014). First metatarsal head osteotomies for the correction of hallux abducto valgus. *Clinics in Podiatric Medicine and Surgery*.

[2] Cassinelli S. J., Herman R., Harris T. G. (2016). Distal Metatarsal Osteotomy for Moderate to Severe Hallux Valgus. *Foot & Ankle International*.

[3] Austin D. W., Leventen E. O. (1981). A new osteotomy for Hallux Valgus: A horizontally directed 'V' displacement osteotomy of the metatarsal head for Hallux Valgus and primus varus. *Clinical Orthopaedics and Related Research*.

[4] Kalish S., McGlamry E. (1974). The modified Keller hallux valgus repair utilizing Silastic implants with some comments on implants in general. *Journal of the American Podiatric Medical Association*.

[5] Donnelly R. E., Saltzman C. L., Kile T. A., Johnson K. A. (1994). Modified Chevron Osteotomy for Hallux Valgus. *Foot & Ankle International*.

[6] Zelen C. M., Young N. J. (2013). Alternative Methods in Fixation for Capital Osteotomies in Hallux Valgus Surgery. *Clinics in Podiatric Medicine and Surgery*.

[7] Jentzsch T., Renner N., Niehaus R. (2018). The influence of the number of screws and additional surgical procedures on outcome in hallux valgus treatment. *Journal of Orthopaedic Surgery and Research*.

[8] Hirvensalo E., Bostman O., Tormala P., Vainionpaa S., Rokkanen P. (1991). Chevron osteotomy fixed with absorbable polyglycolide pins. *Foot & Ankle*.

[9] Pihlajamaki H., Bostman O., Hirvensalo E., Tormala P., Rokkanen P. (1992). Absorbable pins of self-reinforced poly-L-lactic acid for fixation of fractures and osteotomies. *The Journal of Bone & Joint Surgery (British Volume)*.

[10] Small H. N., Braly W. G., Tullos H. S. (1995). Fixation of the Chevron osteotomy utilizing absorbable polydioxanon pins. *Foot & Ankle International*.

[11] Pelto-Vasenius K., Hirvensalo E., Vasenius J., Rokkanen P. (1997). Osteolytic changes after polyglycolide pin fixation in chevron osteotomy. *Foot & Ankle International*.

[12] Böstman O. M., Pihlajamäki H. K. (2000). Adverse tissue reactions to bioabsorbable fixation devices. *Clinical Orthopaedics and Related Research*.

[13] Seitz J. M., Lucas A., Kirschner M. (2016). Magnesium-Based Compression Screws: A Novelty in the Clinical Use of Implants. *The Journal of The Minerals, Metals & Materials Society (TMS)*.

[14] Windhagen H., Radtke K., Weizbauer A. (2013). Biodegradable magnesium-based screw clinically equivalent to titanium screw in hallux valgus surgery: short term results of the first prospective, randomized, controlled clinical pilot study. *Biomedical Engineering Online*.

[15] Plaass C., Ettinger S., Sonnow L. (2016). Early results using a biodegradable magnesium screw for modified chevron osteotomies. *Journal of Orthopaedic Research*.

[16] Plaass C., von Falck C., Ettinger S. (2018). Bioabsorbable magnesium versus standard titanium compression screws for fixation of distal metatarsal osteotomies – 3 year results of a randomized clinical trial. *Journal of Orthopaedic Science*.

[17] Klauser H. (2018). Internal fixation of three-dimensional distal metatarsal I osteotomies in the treatment of hallux valgus deformities using biodegradable magnesium screws in comparison to titanium screws. *Journal of Foot and Ankle Surgery*.

[18] Coughlin M. J., Saltzman C. L., Nunley J. A. (2002). Angular measurements in the evaluation of hallux valgus deformities: A report of the ad hoc committee of the american orthopædic foot & ankle society on angular measurements. *Foot & Ankle International*.

[19] Rahim M. I., Eifler R., Rais B., Mueller P. P. (2015). Alkalization is responsible for antibacterial effects of corroding magnesium. *Journal of Biomedical Materials Research Part A*.

[20] Rau J. V., Antoniac I., Fosca M. (2016). Glass-ceramic coated Mg-Ca alloys for biomedical implant applications. *Materials Science and Engineering C: Materials for Biological Applications*.

[21] Razavi M., Fathi M., Savabi O., Tayebi L., Vashaee D. (2018). Improvement of in vitro behavior of an Mg alloy using a nanostructured composite bioceramic coating. *Journal of Materials Science: Materials in Medicine*.

[22] Hou P., Zhao C., Cheng P. (2017). Reduced antibacterial property of metallic magnesium. *Biomedical Materials*.

[23] Rahim M. I., Rohde M., Rais B., Seitz J., Mueller P. P. (2016). Susceptibility of metallic magnesium implants to bacterial biofilm infections. *Journal of Biomedical Materials Research Part A*.

[24] Hanft J. R., Kashuk K. B., Bonner A. C., Toney M., Schabler J. (1992). Rigid internal fixation of the Austin/Chevron osteotomy with Herbert screw fixation: A retrospective study. *Journal of Foot Surgery*.

[25] Viehe R., Haupt D. J., Heaslet M. W., Walston S. (2003). Complications of screw-fixated chevron osteotomies for the correction of hallux abducto valgus. *Journal of the American Podiatric Medical Association*.

